# Molecular and Pharmacological Characterization of Serotonin 5-HT_2α_ and 5-HT_7_ Receptors in the Salivary Glands of the Blowfly *Calliphora vicina*

**DOI:** 10.1371/journal.pone.0049459

**Published:** 2012-11-08

**Authors:** Claudia Röser, Nadine Jordan, Sabine Balfanz, Arnd Baumann, Bernd Walz, Otto Baumann, Wolfgang Blenau

**Affiliations:** 1 Institute of Biochemistry and Biology, University of Potsdam, Potsdam, Germany; 2 Institute of Complex Systems (ICS-4), Research Center Jülich, Jülich, Germany; 3 Institut für Bienenkunde (Polytechnische Gesellschaft), Goethe University Frankfurt, Oberursel, Germany; University of California, Merced, United States of America

## Abstract

Secretion in blowfly (*Calliphora vicina*) salivary glands is stimulated by the biogenic amine serotonin (5-hydroxytryptamine, 5-HT), which activates both inositol 1,4,5-trisphosphate (InsP_3_)/Ca^2+^ and cyclic adenosine 3′,5′-monophosphate (cAMP) signalling pathways in the secretory cells. In order to characterize the signal-inducing 5-HT receptors, we cloned two cDNAs (*Cv5-ht2α*, *Cv5-ht7*) that share high similarity with mammalian 5-HT_2_ and 5-HT_7_ receptor genes, respectively. RT-PCR demonstrated that both receptors are expressed in the salivary glands and brain. Stimulation of *Cv5-ht2α*-transfected mammalian cells with 5-HT elevates cytosolic [Ca^2+^] in a dose-dependent manner (EC_50_ = 24 nM). In *Cv5-ht7*-transfected cells, 5-HT produces a dose-dependent increase in [cAMP]_i_ (EC_50_ = 4 nM). We studied the pharmacological profile for both receptors. Substances that appear to act as specific ligands of either Cv5-HT_2α_ or Cv5-HT_7_ in the heterologous expression system were also tested in intact blowfly salivary gland preparations. We observed that 5-methoxytryptamine (100 nM) activates only the Cv5-HT_2α_ receptor, 5-carboxamidotryptamine (300 nM) activates only the Cv5-HT_7_ receptor, and clozapine (1 µM) antagonizes the effects of 5-HT *via Cv*5-HT_7_ in blowfly salivary glands, providing means for the selective activation of each of the two 5-HT receptor subtypes. This study represents the first comprehensive molecular and pharmacological characterization of two 5-HT receptors in the blowfly and permits the analysis of the physiological role of these receptors, even when co-expressed in cells, and of the modes of interaction between the Ca^2+^- and cAMP-signalling cascades.

## Introduction

The salivary glands of the blowfly *Calliphora vicina* are an established model system for studying cellular signal transduction processes [1], [2]. In this non-innervated gland, the biogenic amine serotonin (5-hydroxytryptamine; 5-HT) acts as a neurohormone and stimulates fluid secretion [3], [4]. Stimulation of taste receptors on the labellum likely leads to the release of 5-HT into the hemolymph by neurons that have a large soma in the subesophageal ganglion and varicose terminals in a neurohemal plexus on the dorsal surface of the thoracico-abdominal ganglion [4]–[6]. Exposure of the glands to 5-HT causes a parallel rise in the concentration of cytosolic Ca^2+^ ([Ca^2+^]_i_) and of adenosine 3′,5′-cyclic monophosphate ([cAMP]_i_) in the secretory cells [7], [8]. The increase in [Ca^2+^]_i_ elevates the Cl^-^ permeability of both the basolateral and apical membrane and thus facilitates Cl^-^ movement from the haemolymph into the lumen of the gland [9], [10], whereas cAMP has been shown to stimulate active K^+^ transport into the lumen of the salivary gland [10]. Recent findings suggest that cAMP activates a vacuolar-type H^+^ pump that energizes the luminal membrane to drive an escorting cation/H^+^ antiporter [2], [11]. The simultaneous activation of the two signalling pathways is probably attributable to the activation of two different 5-HT receptor subtypes on the surface of the secretory cells, one receptor subtype being linked to the phospholipase C (PLC) / inositol 1,4,5-trisphosphate (InsP_3_) / Ca^2+^ signalling cascade and the other to the adenylyl cyclase / cAMP signalling pathway [12], [13]. More recent studies on blowfly salivary glands indicate a complex functional crosstalk between the two signalling pathways [14]–[16] and we are only just beginning to understand the molecular basis and physiological significance of such crosstalk. To further this understanding, well-characterized pharmacological tools are required that permit the selective and efficient activation or inhibition of one or the other signalling pathway. In this respect, the manipulation of receptor-dependent signalling would be enormously facilitated once the molecular identity and pharmacological properties of the receptors expressed on blowfly salivary glands are known. However, to date no 5-HT receptor has been cloned and characterized from *C. vicina*.

The majority of 5-HT receptors belong to the superfamily of G-protein-coupled receptors (GPCRs). In contrast to the six GPCR-type 5-HT receptor classes identified in vertebrates [17], there are currently only three classes (5-HT_1_, 5-HT_2_ and 5-HT_7_) identified in insects [17]–[19]. 5-HT_1_ receptors have been shown to couple to inhibitory G-proteins (G_i/o_), which cause a reduction of adenylyl cyclase activity and a subsequent decrease in [cAMP]_i_ [20]–[22]. In contrast, the activation of 5-HT_7_ receptors causes the stimulation of adenylyl cyclase activity and a subsequent increase in [cAMP]_i_ resulting from the activation of stimulatory G-proteins (G_s_) [23], [24]. Finally, members of the 5-HT_2_ receptor class most likely engage G_q/11_ proteins that mediate the PLC-dependent generation of InsP_3_ and a rise of [Ca^2+^]_i_ originating from Ca^2+^ release from the endoplasmic reticulum through InsP_3_-gated channels [19]. Therefore, we can reasonably assume that blowfly salivary glands express 5-HT_2_ and 5-HT_7_ receptors.

In order to test the above hypothesis, we cloned 5-HT receptors from *C. vicina*. Two genes encoding members of the 5-HT_2_ and 5-HT_7_ classes were amplified from blowfly mRNA. Following heterologous expression of the receptors in mammalian cells, we examined the relevant pharmacological profiles and intracellular signalling pathways. Ligands displaying receptor-specific preference(s) were subsequently tested in intact blowfly salivary gland by measuring the changes in the transepithelial potential (TEP). Three 5-HT receptor agonists, i.e, 5-carboxamidotryptamine (5-CT), AS 19 and R(+)-lisuride, selectively activate cAMP-dependent electrical responses suggesting that 5-HT_7_ receptors are exclusively involved. 5-Methoxytryptamine (5-MeOT) is the only 5-HT receptor agonist that preferentially activates Ca^2+^-dependent electrical responses, indicating a higher affinity for 5-HT_2_ than for 5-HT_7_ receptors. Clozapine completely abolishes cAMP-signalling in the gland, whereas methiothepin causes an irreversible inhibition of both cAMP- and Ca^2+^-signalling pathways. These results show that the applied molecular approach is suitable for identifying selective ligands of insect 5-HT_2_ and 5-HT_7_ receptor subtypes. These ligands can now be used to unravel the physiological crosstalk mechanisms between signalling pathways in *C. vicina* salivary gland secretory cells as well as similar systems in other insects.

## Materials and Methods

### Animals

Blowflies (*Calliphora vicina*) were reared at 24–26°C under a 12 h: 12 h light: dark cycle. Imagines at the age of three days to three weeks after emergence were used for the experiments.

### Materials

(2*S*)-(+)-5-(1,3,5-Trimethylpyrazol-4-yl)-2-(dimethylamino)tetralin (AS 19) and cinanserin hydrochloride were purchased from Tocris (Bristol, UK), whereas all other substances were from Sigma (Munich, Germany). AS 19 was dissolved in absolute ethanol. R(+)-lisuride was dissolved in dimethylsulphoxide. All other stock solutions were prepared in water.

### Cloning of Cv5-ht2α and Cv5-ht7 cDNAs

Degenerate primers corresponding to conserved regions of dipteran 5-HT receptors were designed to amplify receptor fragments from *C. vicina*. One set of primers (sense 5′-GTIGGIYTIWTHGTIATGCC-3′; antisense 5′-GCYTTYTGYTCIGTIGCIAC-3′) was deduced from the amino-acid sequences VSL(F/I)VMP in transmembrane segment (TM) 2 and VATEQKA just prior to TM6 of 5-HT_2_ receptors. A second pair of primers (sense 5′-GYGTIGCIBTIYTIGTIATG-3′; antisense 5′-AABGGYTTSCGGAARTC-3′) was deduced from the amino-acid sequences CVA(L/V)LVM in TM2 and DFRKPF just after TM7 of 5-HT_7_ receptors. As a template for polymerase chain reactions (PCRs), cDNA was synthesized from poly(A)^+^ RNA isolated from *C. vicina* brains as described previously [11]. The amplification protocol consisted of: one denaturation step at 94°C for 2.5 min followed by 35 cycles at 94°C for 40 s, annealing at 50°C for 1.5 min (*Cv5-ht2α*) or at 54°C for 30 s (*Cv5-ht7*), elongation at 72°C for 1 min and a final extension at 72°C for 10 min. The PCR products were cloned into pGEM-T vector (Promega, Mannheim, Germany) and sequenced (AGOWA, Berlin, Germany and GATC, Konstanz, Germany). Based on this sequence information, specific primers for ‘rapid amplification of cDNA ends’ (RACE) PCR experiments were designed. The missing 5′-region and 3′-region of *Cv5-ht2α* and *Cv5-ht7* cDNA were amplified in consecutive RACE PCR experiments using the SMART^TM^ RACE cDNA PCR kit (Clontech, Heidelberg, Germany). Finally, the entire coding regions of *Cv5-ht2α* and *Cv5-ht7* were amplified using cDNA synthesized from salivary gland mRNA as the template. Sense and antisense primers were designed to anneal in the 5′- and 3′-untranslated region, respectively. The following primers were used: *Cv5-ht2α* sense: 5′-CACAGCTTGTCAAACAGC-3′, *Cv5-ht2α* antisense: 5′-CCCTTCTTCACTTACTTACCTTTCAC-3′; *Cv5-ht7* sense: 5′-GAATCATTAAACACATACACACACCAC-3′, *Cv5-ht7* antisense: 5′-CCAACCTCGACCCTTTATAATACC-3′. The nucleotide sequences of *Cv5-ht2α*, a short splice variant of *Cv5-ht2α*, and *Cv5-ht7* have been submitted to the European Bioinformatics Institute (EBI) database (accession nos. HE657271, HE856266, and HE657272, respectively).

### Multiple sequence alignments and phylogenetic analysis

Amino-acid sequences used for phylogenetic analyses were obtained from the NCBI database. Multiple sequence alignments of the complete amino-acid sequences were performed with ClustalW. Values for identity and similarity were calculated by using the BLOSUM62 substitution matrix in BioEdit 7.0.8 [25]. MEGA 5 [26] was used to calculate the genetic distances between the core sequences and to construct neighbour-joining trees with 2,000-fold bootstrap re-sampling. *Drosophila melanogaster* ninaE (rhodopsin 1) and FMRFamide receptor were used as out-groups.

### RT-PCR amplification of Cv5-ht2α and Cv5-ht7 fragments

Total RNA was isolated with TRIZOL LS (Invitrogen, Karlsruhe, Germany) from brains, flight muscles, Malpighian tubules and salivary glands of adult flies. Samples were digested with DNase I (Ambion, Huntingdon, UK). For negative controls, samples were treated with DNase I and an RNase cocktail (Ambion). *Cv5-ht2α*- and *Cv5-ht7*-specific fragments of 156 bp and 231 bp, respectively, were amplified by using the SuperScript^®^ One-Step RT-PCR System (Invitrogen) and the following primers: *Cv5-ht2α,* sense 5′-CGTTACGCCGCAATAGTCC-3′, antisense 5′-GATGATGATGATGATGCTGAAAGC-3′; *Cv5-ht7,* sense 5′-GCATGCTCTTAATGGAACC-3′, antisense 5′-GAGGCCTTCTTCTCTTTGG-3′. As a control, *C. vicina* β-actin was amplified (sense primer 5′-GGTAATGAACGTTTCCGTTGC-3′, antisense primer 5′-CATACGGAGTATTTGCGTTCTGG-3′). cDNA was synthesized for 30 min at 50°C, followed by a single denaturation step at 94°C for 2 min. Amplification of *Cv5-ht2α*, *Cv5-ht7*, or *Cvactin* fragments was performed for 35 cycles at 94°C for 40 s, 55–60°C for 40 s, and 72°C for 15 s followed by a final extension at 72°C for 10 min.

### Construction of expression vectors

To monitor transfection efficiency and receptor-protein expression, a His-tag was added to the *Cv5-ht2α* cDNA and a haemagglutinin (HA) epitope tag was attached to the 3′ end of the *Cv5-ht7* cDNA. A truncated version of *Cv5-ht2α* cDNA was constructed including the Kozak consensus motif (*CCACC*) [27] immediately 5′ to an ATG-codon preceding the TM1 of the receptor. The PCR product was amplified with the sense primer 5′-AATGAAGCTT *CCACC*ATGAATCAGTCATTGTACTCTAG-3′ and the antisense primer 5′-TTTTCTAGACCTTTCACAATTACACTTGAGCAA-3′, digested with *Hind*III and *Xba*I. A double-stranded 6×-His-encoding oligonucleotide was annealed from complementary oligonucleotides: sense 5′-AAATCTAGACATCATCACCATCACCACTAAGCGGCCGCTTTTT-3′ and antisense 5′-AAAAAGCGGCCGCTTAGTGGTGATGGTGATGATGTCTAGATTT-3′. This fragment was digested with *Xba*I and *Not*I. The construct encoding Cv5-HT_2α_ together with the 6×-His-encoding oligonucleotide were sub-cloned into the *Hind*III and *Not*I sites of the pcDNA6/myc-His A vector (Invitrogen).

To introduce a unique *Nhe*I restriction site and a Kozak consensus motif preceding the initiating ATG-codon of *Cv5-ht7*, a PCR was performed with the sense primer 5′-TTTGCTAGC *CCACC*ATGGATTCGTTAGTTGAAAAC-3′ and the antisense primer 5′-TTTGAATTCGAGAAAACTTTCCCGGGCACC-3′. The fragment was digested with *Nhe*I and *Eco*RI and sub-cloned into the *Nhe*I and *Eco*RI sites of the pcAm5-ht1A-HA vector [22], which is a modified pcDNA3.1(+) vector (Invitrogen) containing a HA-encoding oligonucleotide prior to a TAA stop codon. All constructs were checked by sequencing.

### Heterologous expression of Cv5-HT_2α_ and Cv5-HT_7_ receptors

We applied a previously established protocol [28] to generate cell lines that constitutively expressed either Cv5-HT_2α_ or Cv5-HT_7_. For Cv5-HT_2α_ expression, human embryonic kidney cells (HEK 293; ECACC, Salisbury, UK) were chosen. For Cv5-HT_7_ expression, we used a cell line that had been transfected with a gene encoding a variant of the A2-subunit of the olfactory cyclic nucleotide-gated ion channel [29] (flpTM cells, provided by Sibion biosciences GmbH, Jülich, Germany). Approximately 8 µg of the respective expression vectors were introduced into ∼4×10^5^ cells by a modified calcium-phosphate method [30]. Stably transfected cells were selected in the presence of the antibiotics Blasticidin (0.1 mg/ml; Cv5-HT_2α_) or G418 (0.8 mg/ml; Cv5-HT_7_). Expression of Cv5-HT_2α_ and Cv5-HT_7_ was monitored by Western blotting with commercially available anti-His antibodies (anti-6x His Epitope Tag; Rockland Immunochemicals Inc, Gilbertsville, USA) and anti-HA antibodies (clone 3F10; Roche, Penzberg, Germany), respectively.

### Functional characterization of Cv5-HT_2α_ and Cv5-HT_7_ receptors

The ability of Cv5-HT_2α_ to induce Ca^2+^ signals in the transfected cell line was measured with the Ca^2+^-sensitive dye Fluo-4. Similarly, Cv5-HT_7_-induced cAMP production was indirectly monitored by the cAMP-dependent activation of the cyclic nucleotide-gated ion channel in flpTM cells (see above). The cyclic nucleotide-gated ion channel is permeable to Ca^2+^ ions and thus an increase in [cAMP]_i_ leads to an influx of Ca^2+^ ions, which can be detected with Fluo-4. Cells were grown in 96-well plates to a density of ∼3×10^4^ (Cv5-HT_2α_) or 1.5×10^4^ (Cv5-HT_7_) and then incubated in extracellular solution (ES in mM: 120 NaCl, 5 KCl, 2 CaCl_2_, 2 MgCl_2_, 10 glucose, 10 HEPES, pH 7.4) containing 3 mM probenecid, 0.02% (w/v) Pluronic^®^ F-127 (Sigma) and 2 µM Fluo-4AM (Invitrogen). After 1 h, the loading solution was substituted for dye-free ES. To inhibit cAMP hydrolysis in Cv5-HT_7_-expressing cells, ES containing 100 µM isobutylmethylxanthine (IBMX) was used. Plates were transferred into a fluorescence reader (FLUOstar Omega; bmg, Offenburg, Germany) and Fluo-4 fluorescence was monitored. The excitation wavelength was 485 nm. Fluorescence emission was detected at 520 nm. Once the basal fluorescence in each well had reached a stable value, various concentrations of the respective ligands were added. The resulting changes in Fluo-4 fluorescence were recorded automatically.

The Cv5-HT_7_-expressing cell line was also used to determine receptor-induced cAMP production in response to various biogenic amines directly. Assays to quantify cAMP amounts were performed as described earlier [31] by using the cAMP-Screen System (Applied Biosystems, Darmstadt, Germany).

### Recordings of the transepithelial potential

The abdominal portions of salivary glands were dissected in physiological saline (in mM: 128 NaCl, 10 KCl, 2 CaCl_2_, 2.7 MgCl_2_, 3 Na-glutamate, 2.8 maleic acid, 10 D-glucose and 10 Tris-HCl, pH 7.2). Measurements of transepithelial potential (TEP) were performed by the oil-gap method [32] with a DPA-2F differential amplifier (npi Electronic Instruments, Tamm, Germany). Data were sampled and digitized at 1 Hz (KUSB-3102 module for AD-conversion; Keithley, Germering, Germany) by using the software TestPoint (v4.1; Capital Equipment Corp, Billerica, MA, USA) for data acquisition and storage.

## Results

### Structural properties of Calliphora vicina 5-HT_2α_ and 5-HT_7_ receptors

A PCR strategy was applied to clone full-length cDNAs encoding 5-HT_2α_ and 5-HT_7_ receptors from *C. vicinia*. The nucleotide sequence of *Cv5-ht2α* consists of 4,114 bp. The longest open reading frame (ORF) comprises 3,615 bp and codes for a protein of 1,204 amino-acid residues (Cv5-HT_2α_, Fig. S1) with a calculated molecular mass of 134.1 kDa. Upstream of the translation initiation codon (ATG, position 214–216), stop codons are found in all reading frames. In addition to this cDNA, we amplified a shorter fragment harbouring an ORF of 2,967 bp. The nucleotide sequences of both cDNAs were identical, except for a fragment encoding part of the third intracellular loop (IL) of the receptor that was missing in the shorter variant (Fig. S1, red letters). The smaller transcript most probably originated from alternative splicing, because splice donor and acceptor sites flanked the missing exon.

The deduced amino-acid sequence of Cv5-HT_2α_ consists of a long extracellular N-terminus of 532 residues, seven hydrophobic transmembrane regions (TM) and a short intracellular C-terminal domain (Fig. S1). The N-terminal region harbours 17 consensus sites for aspargine-linked (N-)glycosylation. The IL3 between TM5 and TM6 contains ten consensus sites for phosphorylation by protein kinase C (PKC; S/T-X-R/K) and two consensus sites for phosphorylation by protein kinase A (PKA; R-R/K-S/T). In the IL3 of the shorter variant, only three sites and one site for phosphorylation by PKC and PKA, respectively, are present. Additional PKC phosphorylation sites are present in the IL2 between TM3 and TM4 and in the C-terminal region.

The nucleotide sequence of *Cv5-ht7* comprises 2,363 bp. The longest ORF covers 2,001 bp, coding for a protein of 666 amino-acid residues and a calculated molecular mass of 73.1 kDa (Cv5-HT_7_, Fig. S2). Stop codons are present in all reading frames upstream of the ATG-codon (position 146–148). A computer-based structural analysis of Cv5-HT_7_ (Protscale; http://web.expasy.org/protscale) predicts eight TM segments from which the first (TM0; Fig. S2) is located in the N-terminal segment of the receptor. The following seven TM segments share cognate sequence motifs with those of GPCRs belonging to the 5-HT-receptor sub-family (see Fig. S2) and have thus been termed TM1 to TM7. The N-terminal region of Cv5-HT_7_ harbours 11 consensus sites for N-glycosylation. One consensus site for phosphorylation by PKC is present each in the IL2 and in the C-terminal region.

**Figure 1:**
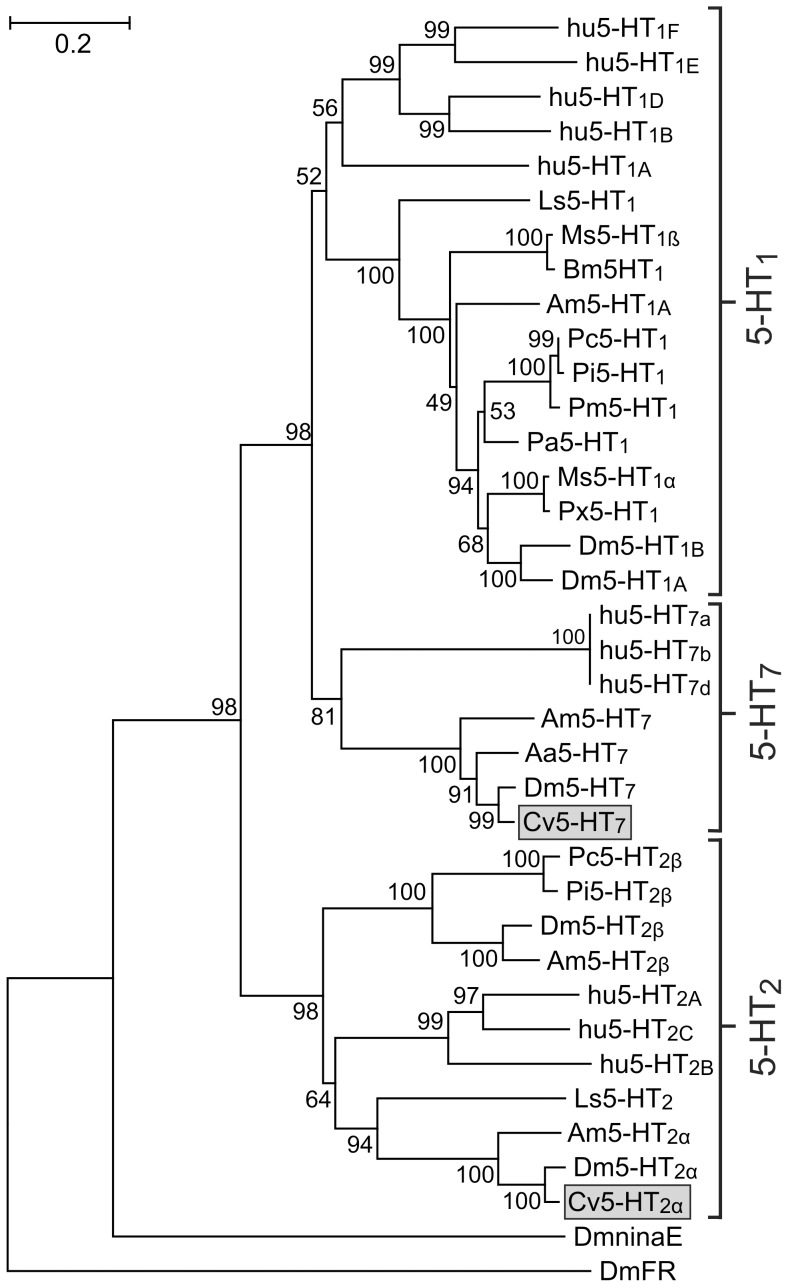
Phylogenetic analysis of Cv5-HT_2α_, Cv5-HT_7_ and 5-HT receptors from various species. Alignments were performed with BioEdit (version 7.0.8) by using the core amino-acid sequences without the variable regions of the N- and C-terminus and the IL3. Genetic distance was calculated with MEGA 5. Receptor sequences and their accession numbers are listed in the order illustrated: human (hu)5-HT_1F_ (NP000857), hu5-HT_1E_ (NP000856), hu5-HT_1D_ (NP000855), hu5-HT_1B_ (NP000854), hu5-HT_1A_ (NP000515), *Lymnaea stagnalis* (Ls)5-HT_1_ (AAA29290), *Manduca sexta* (Ms)5-HT_1ß_ (DQ840516), *Bombyx mori* (Bm)5-HT_1_ (CAA64862), *Apis mellifera* (Am)5-HT_1A_ (CBI75449), *Procambarus clarkii* (Pc)5-HT_1_ (ABX10973), *Panulirus interruptus* (Pi)5-HT_1_ (AAS18607), *Penaeus monodon* (Pm)5-HT_1_ (AAV48573), *Periplaneta americana* (Pa)5-HT_1_ (CAX65666), Ms5-HT_1α_ (ABI33826, *Papilio xuthus* (Px)5-HT_1_ (BAD72868), Dm5-HT_1B_ (CAA77571), Dm5-HT_1A_ (CAA77570), hu5-HT_7a_ (NP000863), hu5-HT_7b_ (NP062874), hu5-HT_7d_ (NP062873), Am5-HT_7_ (CAJ28210), *Aedes aegypti* (Aa)5-HT_7_ (AAG49292), Dm5-HT_7_ (AAF57104), *Calliphora vicina* (Cv)5-HT_7_ (this study, CCF77366), Pi5-HT_2β_ (AAS57919), Pc5-HT_2ß_ (ABX10972), Dm5-HT_2ß_ (AAN13390), Am5-HT_2β_ (CBX90121), hu5-HT_2A_ (NP000612), hu5-HT_2C_ (NP000859), hu5-HT_2B_ (NP000858), Ls5-HT_2_ (ACC16969), Am5-HT_2α_ (CBX90120), Dm5-HT_2α_ (CAA57429), Cv5-HT_2α_ (this study, CCF77376), *D. melanogaster* ninaE-encoded rhodopsin 1 (DmninaE; AFF55712) and *D. melanogaster* FMRFamide receptor (DmFR; AAF47700). The numbers at the nodes of the branches represent the percentage bootstrap support for each branch. The scale bar allows conversion of branch lengths to genetic distance between clades.

BLAST searches of the NCBI database with Cv5-HT_2α_ and Cv5-HT_7_ revealed that both proteins shared homology with insect and mammalian 5-HT receptors. The highest values of sequence conservation were observed between the Cv5-HT_2α_ and 5-HT_2_ receptors of *Drosophila melanogaster* (Dm5-HT_2α_, CG1056; identity 36% / similarity 46%) and *Apis mellifera* (Am5-HT_2α_; 25% / 30%). Similar observations were made for Cv5-HT_7_ and 5-HT_7_ receptors from *D. melanogaster* (Dm5-HT_7_, CG12073; 58% / 64%), *Aedes aegypti* (Aa5-HT_7_; 47% / 55%) and *A. mellifera* (Am5-HT_7_; 37% / 45%). A phylogenetic tree was calculated from a multiple sequence alignment of Cv5-HT_2α_, Cv5-HT_7_, various protostomian 5-HT receptors and human 5-HT receptors (Fig. 1). Both *C. vicina* receptors assembled in clades harbouring members of the respective receptor classes, i.e, 5-HT_2_ and 5-HT_7_.

### Expression patterns of Cv5-ht2α and Cv5-ht7 mRNA

**Figure 2:**
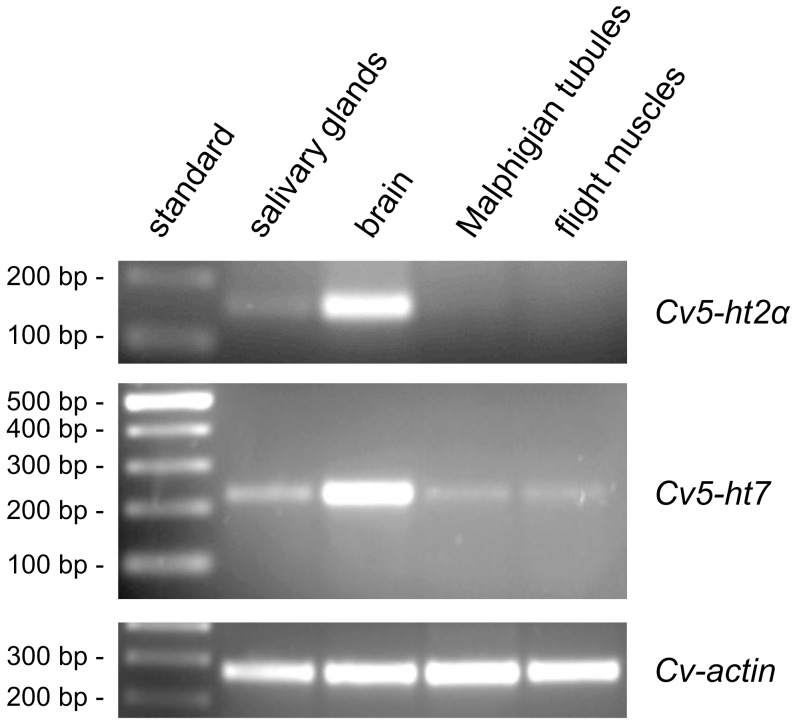
Tissue-specific expression of *Cv5-ht2α* and *Cv5-ht7* mRNA. Receptor-specific fragments were amplified by RT-PCR on RNA samples obtained from various *Calliphora* tissues. Expression of both receptor mRNAs was highest in brain tissue. *Cv5-ht2α* expression was also detected in salivary glands. The *Cv5-ht7* transcript was also detected in flight muscles, Malpighian tubules, and salivary glands. No amplification products were obtained when samples were digested with an RNase cocktail prior to RT-PCR (not shown).

The expression patterns of *Cv5-ht2α* and *Cv5-ht7* mRNA in various tissues of adult flies were examined by RT-PCR. Receptor-specific primer pairs were used to amplify fragments from the IL3 of the receptors. In case of *Cv5-ht2α*, primers were selected for amplification of a fragment common to both splice variants. Whereas *Cv5-ht7* transcripts were detected in all tissues examined, i.e, brain, flight muscles, Malpighian tubules and salivary glands, *Cv5-ht2α* expression was restricted to brain and salivary glands (Fig. 2). According to this semi-quantitative analysis, the largest amount of both receptor mRNAs was present in brain tissue.

### Functional analyses of heterologously expressed Cv5-HT_2α_ and Cv5-HT_7_

To unravel the intracellular signalling pathway(s) and pharmacological properties of both *C. vicina* receptors, we established cell lines that constitutively expressed either Cv5-HT_2α_ or Cv5-HT_7_. However, all attempts to express full-length Cv5-HT_2α_ failed. We reasoned that this negative result was attributable to the extremely long N-terminal segment of Cv5-HT_2α_ (see Fig. S1) and we thus deleted 468 residues of the N-terminus. Use of M_469_ located 64 amino-acid residues upstream of TM1 as a novel protein start site, however, resulted in successful expression of the receptor. Truncation of the N-terminal region was also previously reported to improve expression of *D. melanogaster* Dm5-HT_1A_ and Dm5-HT_1B_ [20]. In contrast to Cv5-HT_2α_, the full-length construct encoding Cv5-HT_7_ could be successfully expressed in the cell line.

**Figure 3:**
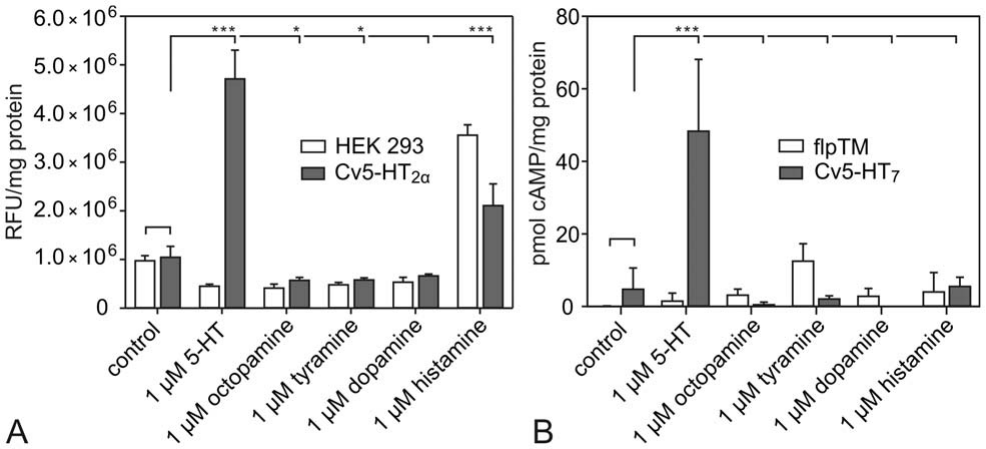
Modulation of intracellular Ca^2+^ and cAMP levels in cell lines constitutively expressing Cv5-HT_2α_ or Cv5-HT_7_. (A) Effect of various biogenic amines on intracellular Ca^2+^ levels in Cv5-HT_2α_-expressing cells. Ca^2+^ levels were determined with Fluo-4. When 1 µM 5-HT, dopamine, tyramine or octopamine was applied, only 5-HT led to an increase in [Ca^2+^]_i_. In both non-transfected and Cv5-HT_2α_-expessing cells, 1 µM histamine stimulated Ca^2+^ signals because of endogenous histamine receptors present in HEK 293 cells. In conclusion, Cv5-HT_2α_ specifically responded to 5-HT with an increase in [Ca^2+^]_i_. (B) Effect of biogenic amines on intracellular cAMP levels in Cv5-HT_7_-expressing cells in the presence of 100 µM IBMX. When 1 µM 5-HT, dopamine, tyramine or octopamine was applied, only 5-HT led to a rise of [cAMP]_i_. In conclusion, Cv5-HT_7_ specifically responded to 5-HT with an increase in [cAMP]_i_. Data are the means±SD of 3 to 8 replicates. Statistically significant differences are indicated by asterisks above the bars (one-way ANOVA followed by Dunnett's multiple comparison test). ***, P < 0.001; **, P < 0.01; *P < 0.05.

To test the ligand specificity of both receptors, various biogenic amines were examined for their ability to elevate [Ca^2+^]_i_ and [cAMP]_i_ in Cv5-HT_2α_- and Cv5-HT_7_-expressing cells, respectively. Non-transfected cells were used as control. In the absence of ligands, basal [Ca^2+^]_i_ was similar in non-transfected and Cv5-HT_2α_-expressing cells (Fig. 3A). Likewise, we did not detect a significant [cAMP]_i_ increase in Cv5-HT_7_-expressing cells in comparison with control cells [cAMP]_i_ (Fig. 3B). When 1 µM 5-HT, dopamine, tyramine or octopamine was applied, only 5-HT led to an increase of [Ca^2+^]_i_ in Cv5-HT_2α_-expressing cells and to a rise of [cAMP]_i_ in Cv5-HT_7_-expressing cells. In both non-transfected and Cv5-HT_2α_-expessing cells, 1 µM histamine stimulated Ca^2+^ signals because of endogenous histamine receptors present in HEK 293 cells [33]. We thus conclude that Cv5-HT_2α_ and Cv5-HT_7_ specifically respond to 5-HT and that Cv5-HT_2α_ activation elicits an increase in [Ca^2+^]_i_, whereas Cv5-HT_7_ activation causes an elevation in [cAMP]_i_.

### Pharmacological characterization of heterologously expressed Cv5-HT_2α_ and Cv5-HT_7_

**Figure 4:**
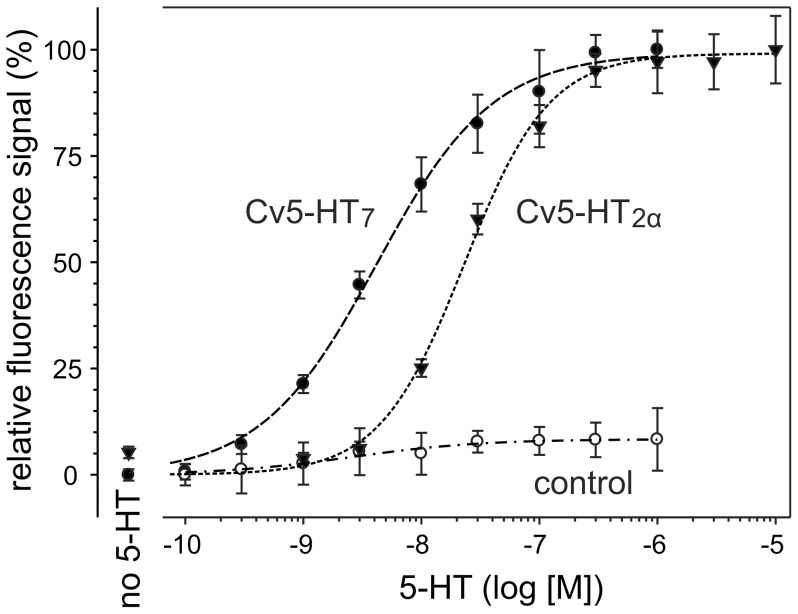
Dose-dependent effects of serotonin (5-HT) on Ca^2+^ signals. Experiments were performed with Cv5-HT_2α_-expressing HEK 293 cells, Cv5-HT_7_-expressing flpTM cells, and non-transfected flpTM cells (control). Increasing concentrations of 5-HT were added to all cell lines and the Ca^2+^-dependent Fluo-4 signal was registered. Data are displayed as the means±SD of eight replicates of a representative experiment. The relative fluorescence signal (%) was normalized to the maximal value ( = 100%) measured at saturating 5-HT concentrations for the respective receptor. For both receptor-expressing cell lines, responses were concentration-dependent and saturable. EC_50_ values of 24 nM and 4 nM were calculated for Cv5-HT_2α_ and Cv5-HT_7_, respectively.

To characterize the pharmacological properties of Cv5-HT_2α_ and Cv5-HT_7_ in more detail, both cell lines were incubated with increasing concentrations of 5-HT. Their responses were concentration-dependent and saturable. Figure 4 shows the normalized dose-response curves for both receptors. Based on these data, EC_50_ values of 24 nM and 4 nM were calculated for Cv5-HT_2α_ and Cv5-HT_7_, respectively.

**Figure 5:**
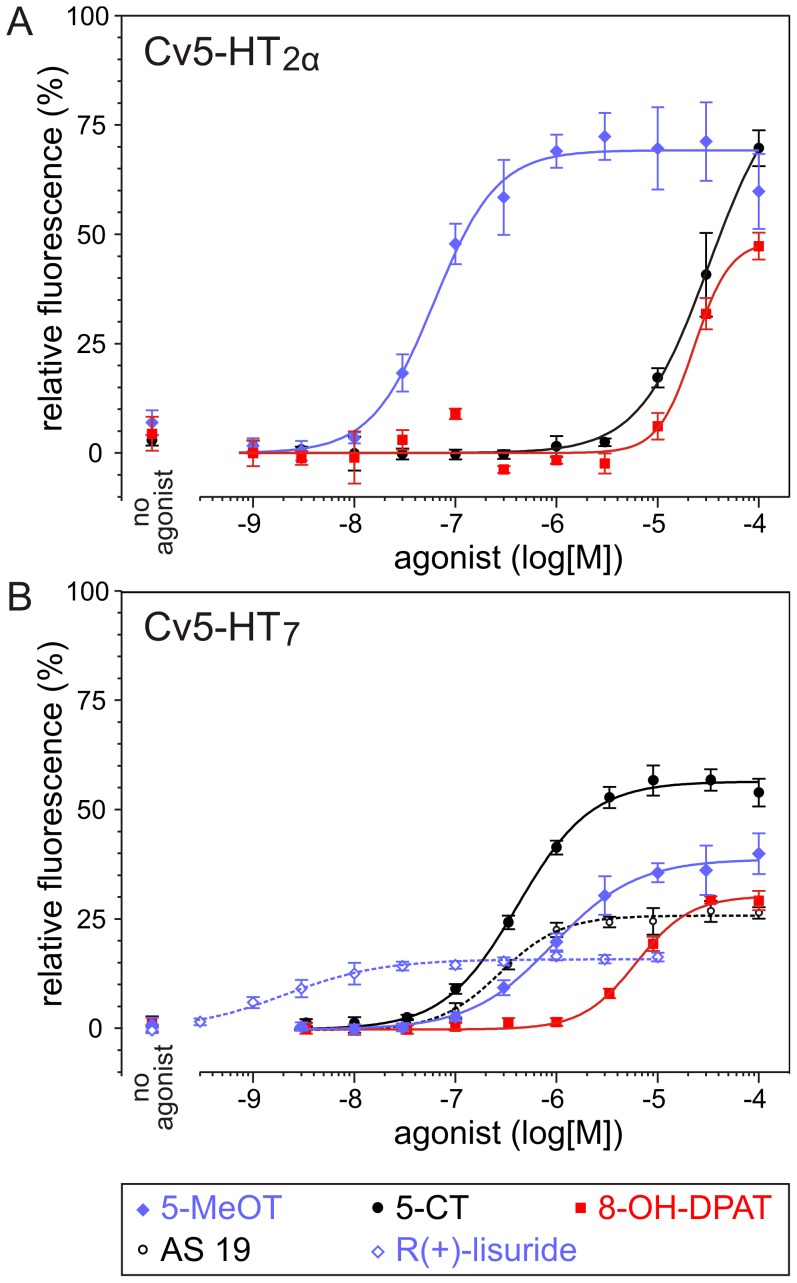
Dose-dependent effects of putative 5-HT receptor agonists on Ca^2+^-dependent Fluo-4 signals in Cv5-HT_2α_-expressing and Cv5-HT_7_-expressing cell lines. Increasing concentrations of 5-HT receptor agonists were added to receptor-expressing cell lines and Ca^2+^-dependent Fluo-4 signals were registered. Data represent the means±SD of four to eight replicates. Fluorescence values were normalized to the maximal value taken from the corresponding dose-response curve for 5-HT ( = 100%). (A) Only 5-methoxytryptamine (5-MeOT), 5-carboxamidotryptamine (5-CT) and (+/−)-8-hydroxy-2-(dipropylamino)tetralin (8-OH-DPAT) activated Cv5-HT_2α_. The potency of 5-MeOT (EC_50_ = 67 nM) was comparable to that of 5-HT (EC_50_ = 24 nM). (B) All tested agonists evoked responses in Cv5-HT_7_-expressing cells. EC_50_ values of all compounds, except for 5-MeOT, were higher at Cv5-HT_2α_ than at Cv5-HT_7_. Compared to 5-HT the efficacy of all agonists at Cv5-HT_7_ is reduced. For example, R(+)-lisuride is the agonist with highest potency but shows very low efficacy. Only a selection of the tested agonists is shown in this figure. EC_50_ values of all tested agonists are given in Table 1.

The ability of both receptors to respond to six established 5-HT receptor agonists was subsequently examined. An overview of the results is displayed in Figure 5 and Table 1. All compounds evoked responses in Cv5-HT_7_-expressing cells, whereas only 5-methoxytryptamine (5-MeOT), 5-carboxamidotryptamine (5-CT) and (+/−)-8-hydroxy-2-(dipropylamino)tetralin (8-OH-DPAT) activated Cv5-HT_2α_. With an EC_50_ of 67 nM, 5-MeOT activation of Cv5-HT_2α_ was comparable to 5-HT. With EC_50_ values of 51 µM and 62 µM, respectively, both 5-CT and 8-OH-DPAT were almost three orders of magnitude less potent than 5-HT or 5-MeOT at Cv5-HT_2α_. As observed for 5-HT, the EC_50_ values of all compounds, except for 5-MeOT, were lower at Cv5-HT_7_ than at Cv5-HT_2α_. The rank order of agonist potency at Cv5-HT_7_ was: 5-HT ≈ R(+)-lisuride >> AS 19 > 5-CT > methysergide > 5-MeOT >> 8-OH-DPAT (see Table 1). However, responses produced by the highest concentrations of these agonists (in particular of R(+)-lisuride) were consistently weaker than the 5-HT-evoked responses (Fig. 5B). Thus, compared to 5-HT the efficacy of all agonists at Cv5-HT_7_ is reduced.

**Table 1:** Agonist profiles of heterologously expressed Cv5-HT_2α_ and Cv5-HT_7_ receptors.

agonist	Cv5-HT_2α_ EC_50_ (µM)	Cv5-HT_2α_ log EC_50_	Cv5-HT_7_ EC_50_ (µM)	Cv5-HT_7_ log EC_50_
5-HT	0.024	−7.62±0.03	0.004	−8.39±0.03
5-MeOT	0.067	−7.17±0.05	0.95	−6.02±0.04
5-CT	51	−4.29±0.05	0.43	−6.37±0.02
8-OH-DPAT	62	−4.21±0.11	7.9	−5.10±0.04
R(+)-lisuride	no effect		0.002	−8.75±0.05
AS 19	no effect		0.30	−6.52±0.04
methysergide	no effect		0.77	−6.11±0.09

**Figure 6:**
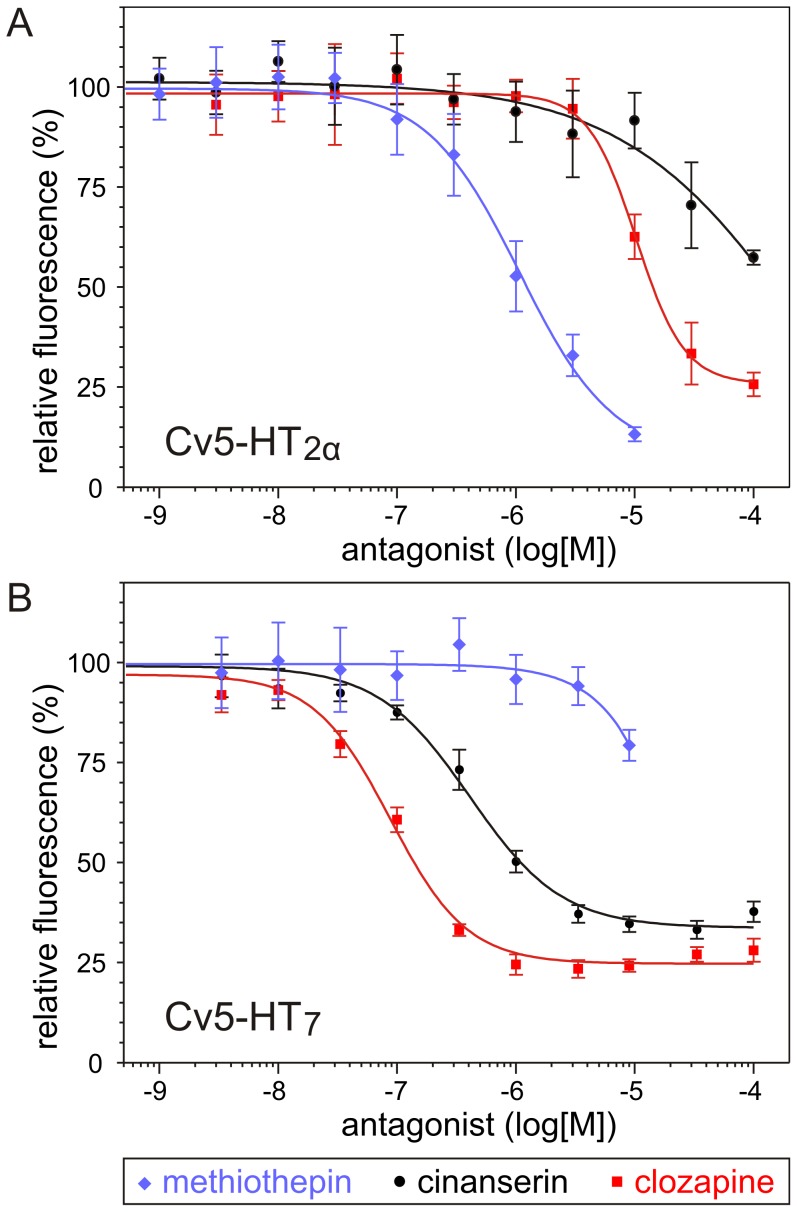
Inhibition of serotonin (5-HT)-induced Ca^2+^ signals in Cv5-HT_2α_- and Cv5-HT_7_-expressing cell lines. Changes in Ca^2+^-dependent Fluo-4 signals were registered automatically. Data represent the means±SD of eight replicates. (A) Cells expressing Cv5-HT_2α_ were incubated with increasing concentrations of the 5-HT receptor antagonists methiothepin, clozapine and cinanserin in the presence of 100 nM 5-HT. Relative fluorescence (%) is normalized to the values obtained with 100 nM 5-HT in the absence of antagonists. From the antagonists shown, methiothepin displayed the highest potency (IC_50_ = 1.2 µM). Methiothepin also showed highest efficacy for suppressing Cv5 HT_2α_-mediated Ca^2+^ signals from all antagonists tested in this study. (B) Cv5-HT_7_-expressing cells were incubated with increasing concentrations of methiothepin, clozapine and cinanserin in the presence 6 nM 5-HT. Relative fluorescence (%) is normalized to the values obtained with 6 nM 5-HT in the absence of antagonists. From the antagonists shown, clozapine displayed the highest potency at Cv5-HT_7_ (IC_50_ = 80 µM). Compared with all other compounds tested in this study, clozapine also displayed the highest inhibitory efficacy (∼77%) at Cv5-HT_7_. IC_50_ values of all antagonists tested in this study are given in Table 2.

We also assayed the effects of 5-HT receptor antagonists to suppress the activation of both *C. vicina* receptors (Fig. 6 and Table 2). Except for spiperone at Cv5-HT_2α_ and methysergide at Cv5-HT_7_, all other compounds inhibited 5-HT-induced responses in the respective cell lines. Values for the half-maximal inhibition (IC_50_) of 5-HT-induced responses were calculated from the dose-response curves. The most potent inhibitors at Cv5-HT_2α_ were mianserin, methiothepin, cyproheptadine and yohimbine with similar IC_50_ values of 0.73, 1.2, 1.6 and 2.9 µM, respectively. The highest efficacy for suppressing Cv5-HT_2α_-mediated Ca^2+^ signals in the cell line was observed for methiothepin followed by clozapine, mianserin and cyproheptadine. Except for yohimbine and methiothepin, all IC_50_ values determined for Cv5-HT_7_ were lower than those for Cv5-HT_2α_. The most potent antagonist at Cv5-HT_7_ was SB-269970 (IC_50_ value 9 nM) followed by mianserin, cyproheptadine and clozapine with IC_50_ values of 67, 73 and 80 nM, respectively (Table 2). Notably, SB-269970, an established 5-HT_7_ receptor antagonist in mammals, displayed the lowest IC_50_ of all compounds tested. However, the efficacy of SB-269970 to reduce the 5-HT-evoked cellular response was small (∼15%). Compared with the other compounds, clozapine displayed the highest inhibitory efficacy (∼77%) at Cv5-HT_7_. In summary, clozapine and spiperone can be considered as selective antagonists of Cv5-HT_7_, whereas methiothepin and methysergide appear selectively to antagonize Cv5-HT_2α_.

**Table 2.** Antagonist profiles of heterologously expressed Cv5-HT_2α_ and Cv5-HT_7_ receptors.

**Table 2:** Antagonist profiles of heterologously expressed Cv5-HT_2α_ and Cv5-HT_7_ receptors.

antagonist	Cv5-HT_2α_ IC_50_ (µM)	Cv5-HT_2α_ log IC_50_	Cv5-HT_7_ IC_50_ (µM)	Cv5-HT_7_ log IC_50_
cinanserin	30	−4.53±0.16	0.40	−6.40±0.04
clozapine	15	−4.82±0.07	0.080	−7.10±0.03
cyproheptadine	1.6	−5.80±0.05	0.073	−7.14±0.08
ketanserin	14	−4.85±0.32	1.5	−5.84±0.33
methiothepin	1.2	−5.92±0.06	not calculable	
methysergide	20	−4.71±0.12	agonistic effect	
mianserin	0.73	−6.14±0.05	0.067	−7.17±0.07
phentolamine	198	−3.70±0.58	4.7	−5.33±0.08
SB-269970	53	−4.27±0.30	0.009	−8.06±0.21
spiperone	no effect		0.28	−6.56±0.06
yohimbine	2.9	−5.54±0.28	192	−3.72±0.10

For each antagonist, IC_50_ values were derived from dose-response curves.

### Effects of 5-HT receptor agonists and antagonists on the electrical response of salivary glands

Pharmacological properties and second messenger coupling of both receptors were determined in cell lines that heterologously expressed the proteins. By measuring the transepithelial potential (TEP) of *C. vicina* salivary glands, we examined whether similar pharmacological characteristics could be observed in an intact ‘mini organ’ that expressed both receptor subtypes. Since our aim was selectively to control either Cv5-HT_2α_ or Cv5-HT_7_ signalling in this preparation, we applied those substances that appeared to be receptor-subtype-specific in the previous experiments (see Tables 1 and 2).

**Figure 7:**
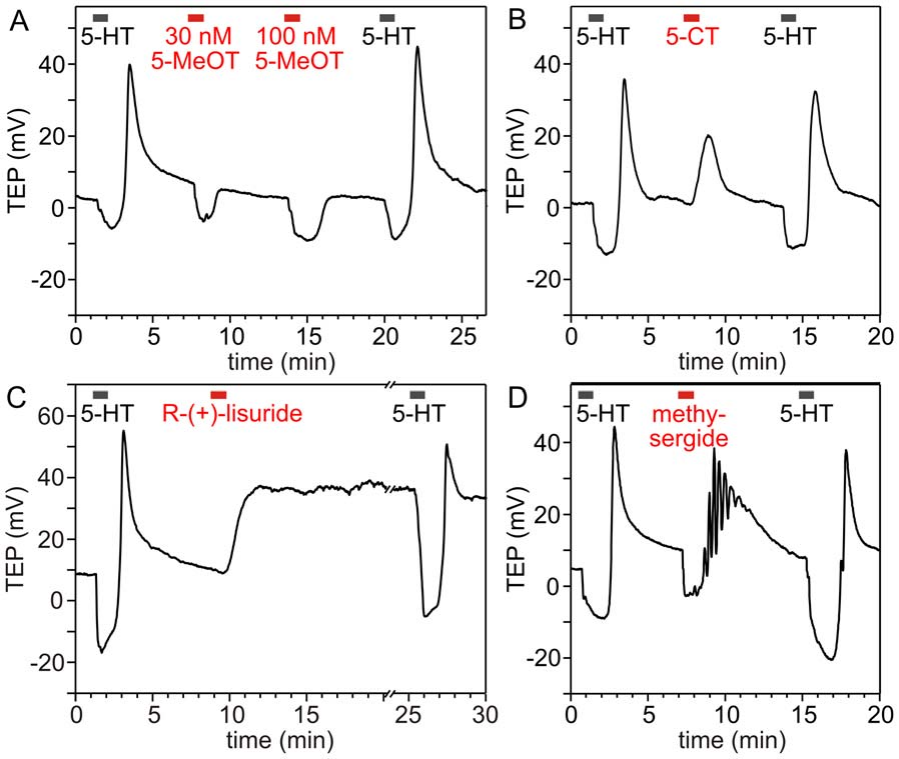
Effect of serotonin (5-HT), 5-MeOT, 5-CT, R(+)-lisuride and methysergide on the transepithelial potential (TEP) of blowfly salivary glands. Application of 30 nM 5-HT resulted in a biphasic change of the TEP, a negative phase during exposure to 5-HT and a positive phase after washout of 5-HT. The negative phase results from Ca^2+^-induced transepithelial Cl^-^ transport into the gland lumen, whereas the positive phase is caused by cAMP-induced cation transport across the apical membrane [9]. It is thus expected that agonists for Cv5-HT_2α_ cause a Ca^2+^-mediated negative-going change of the TEP, whereas agonists for Cv5-HT_7_ elicit a cAMP/PKA-mediated positive-going TEP response. (A) Superfusion of the glands with 30 nM or 100 nM 5-MeOT evoked only a reversible negative-going change of the TEP. Thus, 5-MeOT (when applied at these concentrations) selectively evoked Ca^2+^ signals by activating only Cv5-HT_2α_ in the salivary gland. (B) Application of 300 nM 5-CT induced a reversible positive-going change of the TEP, suggesting that 5-CT selectively generated cAMP signals by activating Cv5-HT_7_. (C) Application of 30 nM R(+)-lisuride induces an irreversible change of the TEP in a positive direction. Thus, R(+)-lisuride selectively and irreversibly activated Cv5-HT_7_. (D) Application of 3 µM methysergide induced a biphasic TEP response, suggesting that methysergide activated Cv5-HT_2α_ and Cv5-HT_7_ in the salivary glands. The oscillations on the rising edge of the methysergide-induced TEP response likely reflect Ca^2+^ oscillations. Note that after washout of methysergide, the negative phase of the 5-HT-induced TEP response was enlarged, whereas the positive phase appeared unchanged. The data shown originate from representative experiments that were replicated independently at least three times.

Brief application of 30 nM 5-HT caused a characteristic biphasic change in the TEP of the glands. In the presence of 5-HT, the TEP went negative. Only after a washout of 5-HT did the TEP go positive and finally decline slowly to the resting value (Fig. 7A) [32]. The negative phase results from Ca^2+^-induced transepithelial Cl^-^ transport into the gland lumen, whereas the positive phase is caused by cAMP-induced cation transport across the apical membrane persisting for a time after 5-HT washout [9]. Application of 30 nM or 100 nM 5-MeOT, the agonist with a higher potency at Cv5-HT_2α_ than at Cv5-HT_7_ (see Fig. 5), induced only a negative-going TEP change, suggesting that 5-MeOT selectively evoked Ca^2+^ signals by activating only Cv5-HT_2α_ in the salivary gland (Fig. 7A). When the gland was stimulated once again with 5-HT after 5-MeOT application, the characteristic biphasic reaction was restored. At higher concentrations (1–100 µM; Table S1), 5-MeOT evoked a biphasic response, although the positive phase was always smaller than the 5-HT-evoked response. Application of 0.3 µM 5-CT induced only a positive-going TEP change (Fig. 7B), suggesting that 5-CT selectively generated cAMP signals by activating Cv5-HT_7_. The kinetics of the positive-going TEP response induced by 0.3 µM 5-CT was similar to that measured after 5-HT washout. At concentrations 1 µM, 5-CT induced a biphasic TEP response (Table S1), suggesting that it activated both 5-HT receptor types. In addition to 0.3 µM 5-CT, AS 19 (1–100 µM) and R(+)-lisuride (30 nM) caused only positive-going changes in the TEP (Table S1). However, the responses to both agonists clearly differed from those to 5-HT or 5-CT. The kinetics of the TEP responses to AS 19 were slow and the amplitude was highly variable (data not shown). Interestingly, treatment of the glands with R(+)-lisuride led to an irreversible positive-going TEP change (Fig. 7C). According to the pharmacological profiles of the two heterologously expressed receptors, methysergide should stimulate Cv5-HT_7_ rather than Cv5-HT_2α_. However, when salivary glands were incubated with 3 µM methysergide, complex biphasic TEP responses were measured (Fig. 7D). During the application of methysergide, the TEP went negative. Only after its washout did the TEP go positive and display oscillations on its rising edge, reminiscent of 5-HT responses at lower 5-HT concentrations. When the glands were subsequently treated with 30 nM 5-HT, a potentiation of the negative phase of the TEP was frequently observed, whereas the positive phase during 5-HT washout appeared unchanged (Fig. 7D). In summary, 5-MeOT at low concentrations (≤0.1 µM) seems to activate selectively Cv5-HT_2α_ in the salivary gland, whereas R(+)-lisuride and 5-CT (0.3 µM) seem to activate selectively and efficiently Cv5-HT_7_, with R(+)-lisuride acting irreversibly and 5-CT reversibly.

**Figure 8:**
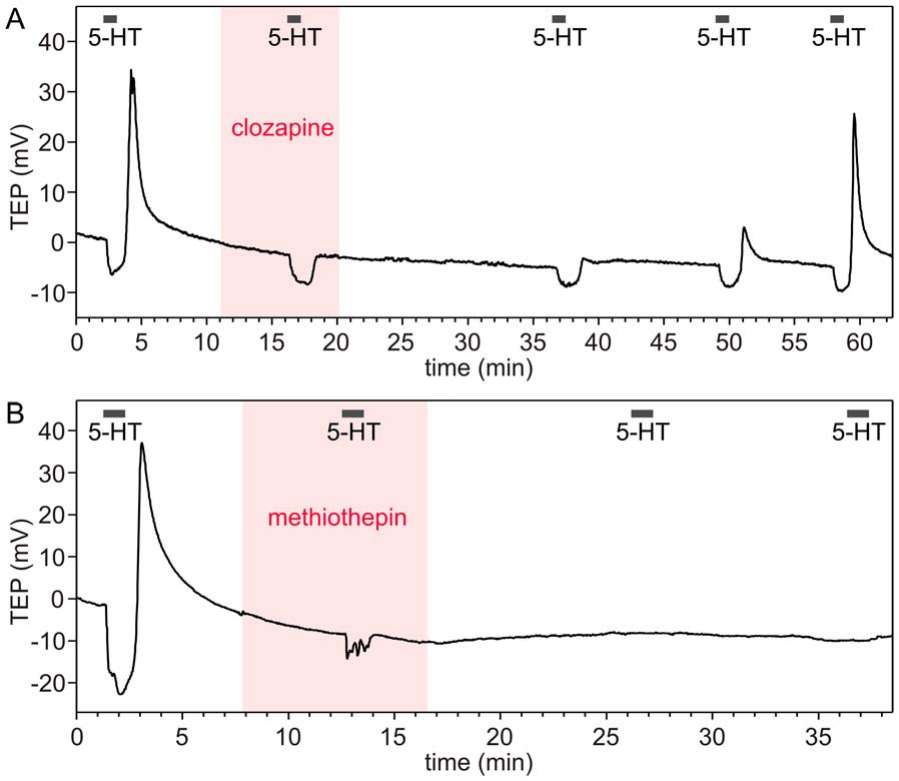
Effects of 5-HT receptor antagonists on serotonin (5-HT)-induced changes of the transepithelial potential (TEP). (A) On a background of 1 µM clozapine, 30 nM 5-HT induced only a negative-going TEP response, suggesting that clozapine specifically antagonizes the effect of 5-HT on Cv5-HT_7_. After wash-out of clozapine, the biphasic response to 5-HT slowly recovered, demonstrating that the clozapine effect was slowly reversible. (B) In the presence of 3 µM methiothepin, 30 nM 5-HT provoked only a small negative TEP phase, and the positive TEP phase was completely abolished, indicating that signalling by both Cv5-HT_2α_ and Cv5-HT_7_ was affected. After washout of methiothepin, the glands were unresponsive to 5-HT. The effects shown could be replicated in at least four independent experiments.

We also tested the antagonistic effects of cinanserin, clozapine, methiothepin and spiperone on 5-HT-induced TEP responses. Clozapine (1 µM) was the only drug that completely and selectively inhibited the positive TEP phase (Fig. 8A), suggesting that it acted as a specific and efficient Cv5-HT_7_ antagonist in the salivary glands. Inhibition was reversible, although a long washout of up to 40 min was necessary to restore the biphasic response to 5-HT. Cinanserin (10 µM) and spiperone (1–10 µM) reversibly but incompletely inhibited the positive phase of the 5-HT-induced TEP response, without any apparent effect on the negative TEP phase (Table S2). Methiothepin (≥1 µM) completely abolished the positive TEP phase; however, the kinetics of the negative phase seemed also to be affected (Fig. 8B). Notably, salivary glands that had been treated once with methiothepin remained unresponsive to subsequent 5-HT stimuli, even after long washout periods. This finding suggests that methiothepin either irreversibly blocks both Cv5-HT_2α_ and Cv5-HT_7_ or has some detrimental side effects in blowfly salivary glands.

## Discussion

The salivary glands of the blowfly *Calliphora vicina* are a well-established model system for studies of cAMP- and Ca^2+^-mediated signalling and for the cellular actions of the biogenic amine 5-HT. The aim of this study was to identify, at the molecular level, the 5-HT receptor subtypes expressed in salivary glands of *C. vicina* and to determine the second messenger pathways activated by these receptors. Moreover, one goal was to discover ligands that permit the selective activation or inhibition of these 5-HT receptor subtypes within the salivary glands.

### Structural and functional features of Cv5-HT_2α_ and Cv5-HT_7_

We cloned two cDNAs coding for 5-HT receptors in *C. vicina*. Based on sequence similarity, one receptor (Cv5-HT_2α_) belongs to the 5-HT_2_ receptor class, whereas the second receptor (Cv5-HT_7_) is a member of the 5-HT_7_ class. On the one hand, both receptors contain key sequence motifs that are important for receptor function, such as the NPxxY motif in TM7, a motif that is involved in receptor activation [34], [35]. The highly conserved Asp-Arg-Tyr (DRY) motif of class A GPCRs is located at the transition of TM3 and IL2, participates in the regulation of conformational states [36], [37] and is conserved in Cv5-HT_7_. In Cv5-HT_2α_, however, the Asp is exchanged for Gly_626_. This substitution is also present in orthologous receptors from *D. melanogaster* (Dm5-HT_2α_, CG1056) [38] and *A. mellifera* (Am5-HT_2α_, CBX90120) and might thus be common for insect 5-HT_2α_ receptors. A cysteine residue (C_1200_) in the C-terminal domain of Cv5-HT_2α_ might serve as target site for palmitoylation. This post-translational modification can create an additional IL that stabilizes the receptor structure [39]. Similarly, a cysteine residue (C_628_) in the C-terminus of Cv5-HT_7_ might become post-translationally palmitoylated.

On the other hand, Cv5-HT_2α_ and Cv5-HT_7_ display some structural peculiarities. The IL3 of GPCRs usually consists of 70–80 amino acids. In Cv5-HT_2α_, this domain comprises 414 amino acids. Notably, the loops of *Lymnea stagnalis* 5-HT_2_ (Ls5-HT_2_) and Dm5-HT_2α_ receptors are of comparable length [38], [40]. Furthermore, both Cv5-HT_2α_ and Cv5-HT_7_ possess large amino-terminal segments of 532 and 276 residues, respectively. This feature is also present in Lym5-HT_2_ and Dm5-HT_2α_. Finally, a hydropathy analysis of Cv5-HT_7_ suggests eight instead of seven TM segments. Similar observations have been described for several aminergic GPCRs of dipteran species, e.g. Dm5-HT_7_ [23], DmTyrR [41] and Aa5-HT_7_ [42], and for a putative tyramine receptor of the cattle tick, *Boophilus microplus* [43]. The function of this additional hydrophobic region in the N-terminal region is largely unknown, although the hydrophobic stretch has been speculated to serve as a signal sequence for post-translational proteolytic cleavage [41], [43].

Our cell-based assays using heterologously expressed Cv5-HT_2α_ and Cv5-HT_7_ demonstrated that both receptors respond specifically to 5-HT with EC_50_ values of 24 nM and 4 nM, respectively. These values are similar to those obtained for orthologous receptors from other protostomes [20], [24], [38], [40], [44], [45]. Experimental support for the assignment of the two receptors to different 5-HT receptor classes was obtained by functional criteria, viz. by the second messenger cascade triggered by receptor activation. Like all 5-HT_2_ receptors, Cv5-HT_2α_ acts *via* the InsP_3_/Ca^2+^ pathway, whereas 5-HT binding to Cv5-HT_7_ induces cAMP formation.

### 5-HT receptors in blowfly salivary glands

**Figure 9:**
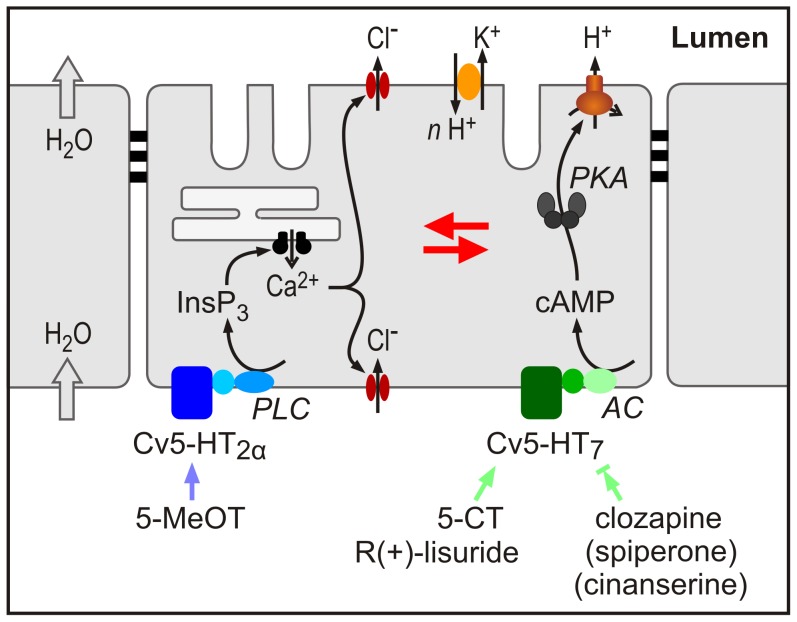
Pharmacological characteristics and signalling pathways of 5-HT receptors in the secretory cells of the blowfly salivary gland. The secretory cells express two types of 5-HT receptors, Cv5-HT_2α_ and Cv5-HT_7_ that can be distinguished pharmacologically. 5-MeOT (100 nM) exclusively activates the Cv5-HT_2α_ receptor, 5-CT (300 nM) and R(+)-lisuride (300 nM) exclusively activate the Cv5-HT_7_ receptor. Clozapine (1 µM) efficiently antagonizes the effects of 5-HT on Cv5-HT_7_, whereas spiperone (10 µM) and cinanserine (10 µM) partially antagonize the effects of 5-HT on Cv5-HT_7_. Cv5-HT_2α_ is linked to the phospholipase C (PLC) / InsP_3_ / Ca^2+^ signalling cascade. The resulting increase in cytosolic [Ca^2+^] [48] enhances the Cl^-^ permeability of the basolateral and apical membrane [9]. Cv5-HT_7_ activation causes stimulation of adenylyl cyclase activity (AC). The rise in [cAMP] leads, *via* protein kinase A (PKA), to an activation of V-ATPase on the apical membrane of the secretory cells [2]. The resulting electrochemical H^+^ gradient energizes K^+^ transport into the lumen of the gland via a putative *n*H^+^/cation exchanger. Red arrows indicate that these signalling pathways influence each other at various levels [14]–[16].

The expression pattern of *Cv5-ht2α* and *Cv5-ht7* was assessed by semi-quantitative RT-PCR. The mRNAs of both receptors are expressed in the brain and in salivary glands, with the highest expression for both genes occurring in brain samples. In addition, *Cv5-ht7*-mRNA but not *Cv5-ht2α*-mRNA was detected in Malpighian tubules and flight muscles. The observation that both receptors are present in blowfly salivary glands supports our previous assumption on the equipment of the secretory cells with 5-HT receptors. Interestingly, according to a recently published microarray dataset (FlyAtlas, http://www.flyatlas.org/, [46]) orthologous receptors are also highly expressed in the salivary gland of *D. melanogaster*. Furthermore, both 5-HT receptor subtypes are likely co-expressed in the secretory cells of blowfly salivary glands, because (1) salivary glands contain only one type of secretory cell, at least by ultrastructural criteria [47] and because (2) all secretory cells within a gland respond to 5-HT with both a rise in cytosolic [Ca^2+^] and the cAMP/PKA-mediated activation of vacuolar-type H^+^-ATPase [48], [49]. Therefore, blowfly salivary glands are well suited for comparing pharmacological and functional properties of heterologously expressed insect 5-HT receptors with those of native receptors (Fig. 9).

In blowfly salivary glands, 1–6 nM 5-HT elicits the half-maximal effect with respect to cAMP formation, electrical response, luminal acidification and fluid secretion [7], [32], [49]. This value is strikingly similar to the EC_50_ value of 4 nM determined for cAMP production *via* heterologously expressed Cv5-HT_7_. In contrast, the production of Ca^2+^ responses in Cv5-HT_2α_-expressing cells (EC_50_ = 24 nM; this study) is approximately an order of magnitude less sensitive to 5-HT than in salivary glands (EC_50_ = 2.8 nM) [48]. This discrepancy might result from N-terminal truncation of the receptor to allow for heterologous expression. Interestingly, a point mutation changing Pro_52_ to Ser in the extended N-terminal segment of the *D. melanogaster* Dm5-HT_2α_ receptor [50] or the complete deletion of the Dm5-HT_2α_ N-terminus has been reported to cause a significant increase rather than decrease in 5-HT affinity [51]. In salivary glands, however, expression of the Cv5-HT_2α_ variant with shortened IL3 may contribute to the observed discrepancy in 5-HT potency. During our efforts to amplify full-length *Cv5-ht2α* cDNA, a fragment corresponding to the shorter variant occurred as a minor constituent when brain cDNA was used but not when salivary gland cDNA was used as template. Therefore, we expressed only the long (major) variant in HEK 293 cells and characterized it pharmacologically. In order to rule out a possible contribution of the short Cv5-HT_2α_ variant to salivary gland physiology it may be worth trying to express this variant in HEK 293 cells and compare the pharmacological profiles of both 5-HT_2α_ receptor variants. Furthermore, the semi-quantitative RT-PCR experiments could be repeated with primer combinations amplifying either the long or the short variant to determine their relative expression levels in different tissues.

In addition, a physical interaction, e.g. dimerization, of Cv5-HT_2α_ and Cv5-HT_7_ (or of Cv5-HT_2α_ and other unidentified GPCRs) in secretory cells might also cause alterations in pharmacological properties. Several studies have shown that homo- or hetero-oligomerization can alter the ligand-binding and signalling properties of GPCRs. For some mammalian 5-HT receptor subtypes, convincing experimental evidence has been presented showing that they can dimerize both in cell culture after heterologous expression and in native tissues [17], [52], [53]. Whether Cv5-HT_2α_ and Cv5-HT_7_ have the ability to form heterodimers remains to be examined in a forthcoming study. Finally, and even more likely, the higher sensitivity of the 5-HT-induced Ca^2+^ response in salivary glands compared with the Cv5-HT_2α_-expressing cell line can result from the biochemical interactions between the second messenger pathways activated by Cv5-HT_2α_ and Cv5-HT_7_ in the secretory cells. Thus, 5-HT concentrations sufficient for Cv5-HT_7_ activation cause a rise in cAMP level and the cAMP/PKA system might sensitize the Cv5-HT_2α_/PLC/InsP_3_/Ca^2+^ cascade. One mode of sensitization might occur at the level of the InsP_3_-induced Ca^2+^ release that is potentiated by PKA activity [14]. Additionally or alternatively, the presence of potential phosphorylation sites for PKA might provide the means for influencing Cv5-HT_2α_ sensitivity in a cAMP/PKA-dependent mode, as demonstrated for other 5-HT receptors [54], [55].

Positive crosstalk mechanisms between the cAMP/PKA and the PLC/InsP_3_/Ca^2+^ signalling pathways activated in parallel in the secretory cells might also account for the differences in the slope of the dose-response curves to 5-HT between native salivary glands and Cv5-HT_2α_- or Cv5-HT_7_-expressing cell lines. In the heterologous expression system, the dynamic range of the dose-response curve to 5-HT extends over about two log units for each receptor subtype. In salivary glands, however, the 5-HT-induced electrical responses, secretory responses and luminal pH changes are confined in their dynamic range to 5-HT concentrations between about 1 to 10 nM [7], [32], [49]. These modes of interplay between signalling pathways activated by 5-HT and the functional implications of the presence of two types of 5-HT receptors will be subject to future investigations.

### Pharmacological characteristics of Cv5-HT_2α_ and Cv5-HT_7_

In the heterologous expression system, 5-MeOT, which is known as a non-selective agonist of mammalian 5-HT receptors [56], was the only substance that displayed a higher affinity for Cv5-HT_2α_ than for Cv5-HT_7_ (see Table 1). These data are consistent with the results of our TEP measurements on blowfly salivary glands. Here, 5-MeOT application induced only a negative-going TEP change at concentrations ≤100 nM. This strongly indicates activation of Cv5-HT_2α_. At higher concentrations, 5-MeOT causes biphasic TEP responses, suggesting co-activation of Cv5-HT_2α_ and Cv5-HT_7_. The use of 5-MeOT for the selective activation of Cv5-HT_2α_ *in vivo* is thus confined to a narrow concentration range. On the other hand, AS 19, R(+)-lisuride and 5-CT were identified as selective agonists of the Cv5-HT_7_ receptor. On blowfly salivary glands, these substances induced only positive-going changes in the TEP, corroborating the results obtained with the heterologously expressed receptors. Whereas AS 19 and 5-CT induced reversible electrical responses in salivary glands, the R(+)-lisuride-evoked response was irreversible. Secretion measurements in *Periplaneta americana* salivary glands have also shown that R(+)-lisuride causes a persistent activation of salivary secretion [57]. These findings suggest that R(+)-lisuride locks the receptor in an almost non-desensitizing state.

Berridge and Heslop [13] have tested the effect of various putative 5-HT receptor antagonists on the rates of both fluid secretion and InsP_3_ production in blowfly salivary glands. Two groups of substances can be distinguished in this classic study. One group of antagonists, including phentolamine, cinanserin and spiperone, seem to act preferentially on 5-HT receptors coupled to cAMP signalling. The other group, including methysergide and yohimbine, preferentially diminish the activity of 5-HT receptors evoking Ca^2+^ signals. Our data using heterologously expressed receptors largely verify and substantiate these observations. Moreover, according to their pharmacological profiles (see Table 2), spiperone, cinanserin and clozapine should act as specific antagonists of Cv5-HT_7_. Surprisingly, only clozapine showed a selective and full inhibition of the cAMP-mediated positive-going TEP response. Even at concentrations sufficient to maximally inhibit 5-HT-evoked effects on heterologously expressed Cv5-HT_7_, spiperone (1 µM) and cinanserin (10 µM) blocked the positive-going TEP change only partially.

In the heterologous expression system, methiothepin is more potent and more efficacious than methysergide with respect to inhibiting Cv5-HT_2α_. Interestingly, whereas methiothepin at high concentrations (10 µM) also has a slight antagonistic effect on Cv5-HT_7_, methysergide activates Cv5-HT_7_ in a concentration range that inhibits Cv5-HT_2α_-mediated responses. When applied to salivary glands, however, methiothepin abolishes both the positive Cv5-HT_7_-mediated TEP response and the negative Cv5-HT_2α_-mediated TEP response irreversibly. An inhibitory effect of methiothepin on 5-HT_7_ receptors has also been shown for orthologous receptors in *Caenorhabditis elegans* [58], *A. mellifera* [24] and humans [59]. When methysergide is applied to salivary glands, it produces a negative TEP phase followed by oscillations that lead to a transient positive phase. This observation suggests that methysergide, in contrast to the situation in the heterologous expression system, has an agonistic effect on both 5-HT_2α_ and 5-HT_7_ receptors in blowfly salivary glands. The finding that pharmacological data obtained from heterologously expressed receptors and physiological effects triggered by 5-HT receptors *in vivo* can differ has also been obtained for the human 5-HT_1D_ receptor [60].

In summary, the pharmacological profiles determined using heterologously expressed Cv5-HT_2α_ and Cv5-HT_7_ represent an important step towards the identification of ligands that can be specifically applied to manipulate signalling pathways *in vivo*. Blowfly salivary glands are an ideal model system for examining the effect and specificity of selected drugs in a native system. Applying this two-step approach, we have been able to show that 5-MeOT at concentrations of up to 100 nM exclusively activates the Cv5-HT_2α_ receptor, whereas 5-CT at concentrations of up to 300 nM exclusively activates the Cv5-HT_7_ receptor. Furthermore, 1 µM clozapine efficiently antagonizes the effects of serotonin on Cv5-HT_7_ in blowfly salivary glands. We envisage that these specific and selective pharmacological tools will greatly facilitate future studies focusing on receptor and signalling pathway crosstalk mechanisms, which now can be addressed with hitherto unprecedented precision.

## Supporting Information

Figure S1 Sequence alignment of Cv5-HT_2α_ and orthologous receptors from *Drosophila melanogaster* (Dm5-HT_2α_, CG1056) and *Apis mellifera* (Am5-HT_2α_, CBX90120). Identical residues are shown as white letters against black, whereas conservative substitutions are shaded in grey. Putative transmembrane domains (TM1-7) are indicated by bars. Potential N-glycosylation sites (inverted filled triangles), putative phosphorylation sites for PKC (filled circles) or PKA (open circles) and the putative palmitoylation site (asterisk) of Cv5-HT_2α_ are labelled. Amino-acids that are absent in a splice variant of Cv5-HT_2α_ are shown in red. The amino-acid position is indicated on the right.
(TIF)

Figure S2 Sequence alignment of Cv5-HT_7_ and orthologous receptors from *Drosophila melanogaster* (Dm5-HT_7_, CG12073), *Aedes aegypti* (Aa5-HT_7_, AF296125) and *Apis mellifera* (Am5-HT_7_, AM076717). Identical residues are shown as white letters against black, whereas conservative substitutions are shaded in grey. Putative transmembrane domains (TM1-7) are indicated by bars. An additional hydrophobic domain (TM0) is present in the N-terminal region of Cv5-HT_7_ and is indicated by a light-grey bar. Potential N-glycosylation sites (inverted filled triangles), putative phosphorylation sites for PKC (filled circles) or PKA (open circles) and the putative palmitoylation site (asterisk) of Cv5-HT_7_ are labelled. The amino-acid position is indicated on the right.
(TIF)

Table S1 Evaluation of the effect of 5-HT receptor agonists on the transepithelial potential (TEP) of blowfly salivary glands. ++ high amplitude, + low amplitude, - no apparent effect, -/+ variable effect
(DOCX)

Table S2 Evaluation of the effect of 5-HT receptor antagonists on the transepithelial potential (TEP) response evoked with 10 nM or 30 nM 5-HT in blowfly salivary glands. ++ complete, + incomplete, - no apparent effect, -/+ variable effect
(DOCX)
