# Supplementary material for: Molecular and Pharmacological Characterization of Serotonin 5-HT2α and 5-HT7 Receptors in the Salivary Glands of the Blowfly Calliphora vicina
Source: PLoS One. 2012 Nov 8;7(11):e49459. doi: 10.1371/journal.pone.0049459 (PMC3493529; doi:10.1371/journal.pone.0049459)
Supplement: Table S1 — Evaluation of the effect of 5-HT receptor agonists on the transepithelial potential (TEP) of blowfly salivary glands. ++ high amplitude, + low amplitude, - no apparent effect, -/+ variable effect (DOCX) [file pone.0049459.s003.docx]

**Table S1**

| **agonist** | **concentration** | **negative TEP phase** | **positive TEP phase** | **reversibility** |
| --- | --- | --- | --- | --- |
| 5-MeOT | 0.03-0.1 µM | ++ | - | yes |
| 5-MeOT | 1-100 µM | ++ | + | yes |
| 5-CT | 0.3 µM | - | + | yes |
| 5-CT | 10 µM | ++ | ++ | yes |
| R(+)-lisuride | 0.03-0.3 µM | - | ++ | no (within 20 min) |
| AS 19 | 100 µM | - | -/+ | yes |
| methysergide | 3 µM | + | +/++ | yes |
