# Supplementary material for: Molecular and Pharmacological Characterization of Serotonin 5-HT2α and 5-HT7 Receptors in the Salivary Glands of the Blowfly Calliphora vicina
Source: PLoS One. 2012 Nov 8;7(11):e49459. doi: 10.1371/journal.pone.0049459 (PMC3493529; doi:10.1371/journal.pone.0049459)
Supplement: Table S2 — Evaluation of the effect of 5-HT receptor antagonists on the transepithelial potential (TEP) response evoked with 10 nM or 30 nM 5-HT in blowfly salivary glands. ++ complete, + incomplete, - no apparent effect, -/+ variable effect (DOCX) [file pone.0049459.s004.docx]

**Table S2**

| **antagonist** | **concentration** | **suppression of the negative TEP phase** | **suppression of the positive TEP phase** | **reversibility** |
| --- | --- | --- | --- | --- |
| clozapin | 1 µM | - | ++ | yes |
| methiothepin | 3 µM | -/+ | ++ | no |
| spiperone | 1-10 µM | - | + | yes |
| cinanserin | 10 µM | - | + | yes |
